# The Alonissos Study: Cross-Sectional Study of the Community Respiratory Health Status in a Greek Healthcare Access Underprivileged Island

**DOI:** 10.3390/healthcare10122338

**Published:** 2022-11-22

**Authors:** Petros Kassas, Eudoxia Gogou, Charalampos Varsamas, Konstantinos Vogiatzidis, Aggeliki Psatha, Maria Pinaka, Dimitra Siachpazidou, Alexandra Sistou, Eleftherios D. Papazoglou, Despoina Kalousi, Konstantina Vatzia, Kyriaki Astara, Nikolaos Tsiouvakas, Sotirios G. Zarogiannis, Konstantinos I. Gourgoulianis

**Affiliations:** 1 Department of Respiratory Medicine, Faculty of Medicine, School of Health Sciences, University of Thessaly, Biopolis, 41110 Larissa, Greece; 2 Department of Physiology, Faculty of Medicine, School of Health Sciences, University of Thessaly, Biopolis, 41500 Larissa, Greece

**Keywords:** chronic obstructive pulmonary disease, insular public health, obstructive sleep apnea syndrome, prevalence, primary healthcare, spirometry

## Abstract

In this study, we investigated the self-reported (questionnaire-based) prevalence of Obstructive Sleep Apnoea Syndrome (OSAS) and the prevalence of Chronic Obstructive Pulmonary Diseases (COPD) in the context of demographics and adherence to the Mediterranean diet in the general population of Alonissos, a non-profit line island in Greece (i.e., with scarce boat transportation to the mainland). In this cross-sectional study, 236 inhabitants of Alonissos participated (circa 10% of the island’s population), and 115 males and 121 females were evaluated with appropriate questionnaires for OSAS, COPD, and adherence to the Mediterranean diet and subsequently underwent spirometry testing to establish COPD diagnosis. The self-reported prevalence of OSAS and COPD was 9.44% and 18.8%, respectively. However, only 8.99% of the participants were diagnosed with COPD based on their spirometry testing. Adherence to the Mediterranean Diet was moderate. The high prevalence of COPD and OSAS in this underprivileged island in terms of healthcare access highlights the need for improvements in health promotion and primary healthcare provision in non-profit line Greek islands.

## 1. Introduction

Greece has approximately 6000 islands out of which 117 are inhabited; however, several are very distant from the mainland and belong to the non-profit line, which means that there is infrequent transport to the mainland [1]. Moreover, their geographical distribution poses disadvantages to healthcare access since they have a small Primary Healthcare Center with very limited resources and minimal staff. Thus, these islanders need to travel to the mainland to undergo standard medical examinations [1]. Additionally, there is a scarcity of epidemiologic data regarding these communities and very little is known about the health status of the inhabitants; thus, no focused health promotion strategies can be applied [2]. On the other hand, two recent studies by our research group highlighted the fact that insular smoking prevalence is substantially higher than the already-high national prevalence [1,3]. In the first study, the smoking prevalence among 700 participants among four Greek insular complexes (Ionian islands, Cyclades, Dodecanese, and Northeast Aegean) was 29.7% [1], and in the second study, the smoking prevalence among 50 participants of the insular complex of Sporades was 54% as compared to 35.78% among 204 participants of mainland Greece [3]. Furthermore, according to the 2021 Health at a Glance OECD Report, Greece ranks fifth in the prevalence of active smokers over the age of 15 with a percentage of 24.9%, which is much higher than the OECD average, which is 16% [4]. The above facts clearly demonstrate a pressing need for the identification of the Greek insular burden of respiratory diseases that are smoking related, including chronic obstructive pulmonary disease, obstructive sleep apnea syndrome, and several cancers.

The aim of the present study was to assess the self-reported prevalence of Chronic Obstructive Pulmonary Disease (COPD) and Obstructive Sleep Apnea Syndrome (OSAS) and their associations with Mediterranean dietary habits in the community of Alonissos island (also written as Alonnisos), a non-profit line Greek island of the Sporades complex.

## 2. Materials and Methods

### 2.1. Study Design

This cross-sectional study was conducted in the Primary Healthcare Center of Alonissos island for the period between 5 July and 11 July 2018. This is the only healthcare provision entity in Alonissos and is located in the capital town of Alonissos, Patitiri. Alonissos belongs to the insular complex of Sporades and is a non-profit line island. According to the 2011 Census, the resident population was 2750 inhabitants [5].

### 2.2. Study Setting and Enrolment

The study was coordinated with the aid of the Alonissos Municipality Community Center, which officially informed the local population regarding the dates and scope of the study via a press release 2 weeks before the start of the study (22 June 2018). The Community Center arranged the appointments of the volunteering participants with the research team that comprised 2 faculty members of the Faculty of Medicine of the University of Thessaly (S.G.Z., K.I.G), 1 general practitioner (E.G), 2 respiratory medicine consultants (H.V., A.P.), 1 cardiologist consultant (K.V.), 3 Nurses (D.S., M.P., A.S.), 1 graduate student (P.K.), and 5 undergraduate students of the Medical Faculty (E.D.P., D.K., K.V., K.A., N.T.).

Upon arrival at the Health Community Center, each participant was thoroughly informed about the purpose of the study and the procedures involved and provided signed informed consent. Subsequently, a nurse obtained the medical history of each participant, questionnaires were provided and answered, and subsequent spirometry testing was performed and evaluated by the respiratory medicine consultant. Each questionnaire had to be fully answered in order to be used for further analysis. Each participant answered the questionnaires in the presence of a member of the research team for any potential clarifications needed. The total number of participants in the study was 245; however, 9 of them were excluded from the analysis as they did not meet the inclusion criteria. Thus, the sample size of the study was 236 individuals. The inclusion criteria were (a) age above 18 years, (b) permanent residence of the participant on the island (≥6 months per year), and (c) possession of a driver’s license.

### 2.3. Assessment Tools-Questionnaires

The assessment tools used in our study were (a) the Berlin questionnaire (BQ) for the evaluation of OSA risk [6], (b) the Epworth Sleepiness Scale (ESS) for the evaluation of daytime sleepiness [7], (c) the COPD Population Screener (COPD-PS) for the evaluation of COPD risk [8], (d) the Mediterranean Diet Score (MedDietScore) for evaluating the adherence to the Mediterranean Diet [9,10], and (e) the demographic data questionnaire for recording the population characteristics.

### 2.4. Assessment Tools—Spirometry

The spirometry procedure was conducted as previously outlined in studies in the community by our research team [11]. More specifically, the spirometry test was performed with a dry spirometer (Spirolab FCC ID: TUK-MIR045) according to the American Thoracic Society’s recommendations [12]. Calibration checks were performed every morning before the beginning of the spirometry program. Spirometry testing was performed by eight physicians and two nurses who had undergone a special training program. Forced expiratory maneuvers were repeated until three acceptable reproducible tests were obtained and the best FEV1, FVC, and FEV1/FVC ratios were recorded. The spirometric reference values used were those proposed by the ERS statement [13]. An acceptable maneuver was defined using the previously described criteria [14].

### 2.5. Diagnosis of COPD

Diagnosis of COPD was established with the presence of a postbronchodilator FEV1/FVC ratio of less than 0.70 following GOLD guidelines [15]. All patients using inhaled medication were instructed to avoid their inhalers for 12 or 24 h depending on the drug formulation (for instance, SABA ≥ 4 h, LABA ≥ 15 h, LAMA ≥ 12 or 24 h) before spirometry. A bronchodilator reversibility test with 400 µg of salbutamol was performed in all patients with baseline prebronchodilator obstructive spirometry. A positive response to bronchodilators was defined as an increase in the FEV1 or FVC of 200 mL and 12% from the prebronchodilator value (baseline value) [11,14,16]. All participants with a positive bronchodilator response were excluded from the analyses. Classification of COPD was based on the postbronchodilator FEV1 (%) according to GOLD guidelines (Stage I/mild COPD, FEV1 ≥ 80%; Stage II/moderate COPD, 50% ≤ FEV1 < 80%; Stage III/severe COPD, 30% ≤ FEV1 < 50%; Stage IV/very severe COPD, FEV1 ≤ 30% or FEV1 < 50% with respiratory failure). Patients with COPD were also allocated into four groups by GOLD (GOLD Groups A, B, C, and D) according to the 2020 GOLD guidelines [15].

### 2.6. Statistical Analysis

Data normality was assessed by the Kolmogorov–Smirnov test. Data are presented as the median with interquartile ranges in parenthesis for skewed data. The Mann–Whitney U test was used for comparisons of continuous data between groups. Categorical variables were expressed as percentages, and the chi-square or Fischer’s exact test was used when comparing the proportions of independent groups. The Wilson–Brown test was used to calculate the performance of the COPD-PS test. Data were tabulated in Excel and statistical analysis was performed with GraphPad Prism v.9.0. *p*-values < 0.05 were considered statistically significant.

## 3. Results

### 3.1. Sample Characteristics

The final sample comprised 236 individuals, of which 115 were male and 121 were female, corresponding to 8.42% of the population of Alonissos island (which was 2804 inhabitants in the 2011 Greek Census). The median age of the sample was 55 (40, 69) years and the median BMI was 26.59 (23.47, 29.76). A high prevalence of smoking was observed as 42% (99/236) of the population were active smokers. A detailed description of the demographic characteristics of the participants based on their gender is provided in Table 1.

### 3.2. COPD High-Risk Detection by COPD-PS and COPD Diagnosis by Spirometry Testing

The COPD-PS was completed by 234/236 participants. Out of the 234 participants, 44 were found to be at high COPD risk (18.8%); 26 were males (23.01%) and 18 (14.88%) were females. High- and low-COPD-risk participants differed significantly in their age (*p* < 0.001), smoking history (*p* = 0.01), and packyears (*p* < 0.001). The odds ratio of participants with a high-risk COPD-PS score to a high-risk BQ score was 2.781 (95%CI; 1.404, 5.782) and to a high-risk ESS score was 3.124 (95%CI; 1.404, 5.782).

Out of the 236 participants, 189 successfully performed spirometry testing. The remaining 47 participants could not complete the spirometry procedure, or their spirometry test was ≤6 s. Out of the 189 participants, 17 were diagnosed with COPD based on GOLD criteria (8.99%) (9/101 males (8.91%) and 8/88 females (9.09%)). Their GOLD classification was GOLD I-8 participants (4.23%), GOLD II-5 participants (2.65%), GOLD III-3 participants (1.59%), and GOLD IV-1 participants (0.53%). Additionally, no correlation was found between those with pathological or normal spirometry and those at high or low OSA risk (*p* = 0.27), high or low sleepiness risk (*p* = 0.57), or high or low OSAS risk (*p* = 0.17). Detailed statistics are provided in Table 2.

### 3.3. Evaluation of COPD PS Tool Based on the Spirometry Test Diagnosis of COPD

We used the results of the spirometry diagnosis of COPD to assess the performance of COPD-PS in our population. We found that the sensitivity of COPD-PS was 0.143 and the specificity was 0.922. The positive predictive value was 0.294, while the negative predictive value was 0.826.

### 3.4. Self-Reported OSAS Risk in Alonissos Population

Regarding the BQ, 233 out of the 236 participants successfully completed it. Out of the 233, 63 were found at high OSA risk (27.09%), of which 34 were male and 29 were female (29.82% and 24.37%, respectively). Statistically significant differences were found between the high- and low-OSA-risk participants concerning their age (*p* < 0.001), BMI (*p* < 0.001), smoking habits (*p* < 0.001), and packyears (*p* = 0.025). OSA risk was not influenced by adherence to the Mediterranean Diet (*p* = 0.20) or gender (*p* = 0.38).

Regarding the ESS, 65 out of the 236 participants, had an ESS score > 10 (27.66%), of which 38 were male (33.04%) and 27 were female (22.50%). Significant differences were found between the participants at high and low Daytime Sleepiness risk, with respect to age (*p* = 0.009) and packyears (*p* = 0.032).

Finally, 22 out of the 232 participants (9.48%) were found at high risk in both BQ and ESS, which qualifies as high risk for OSAS, of which 12 were male (10.53%) and 10 were female (8.40%). High- and low-OSAS-risk participants significantly differed in age (*p* < 0.001), BMI (*p* < 0.001), smoking habits (*p* = 0.032), packyears (*p* < 0.001), and MedDietScore (*p* = 0.035). Details regarding high-risk participants according to BQ, ESS, and their combination are given in Table 3.

## 4. Discussion

Due to the high prevalence of smoking that we had observed in previous studies in the Greek islands, we aimed at investigating the level of respiratory health in the community of Alonissos, an underprivileged non-profit line island of the Sporades Island complex. In our study, nearly 8.5% of the total Alonissos population participated, and 42% of the participants were active smokers, a very high prevalence. Furthermore, 30% of the participants were ex-smokers and only 28% have never smoked, thus the smoking burden on the island is very pronounced. According to the latest study regarding smoking prevalence in Greece performed in 2020, 28% of the population are active smokers, so the numbers remain very high [17]. In Central Greece and Thessaly, the prevalence is even higher, ranging from 32.4 to 35.78% [1,3,18]. These numbers are very alarming since smoking is the cause of COPD, of which the burden in Greece is significant [19]. COPD is substantially underdiagnosed, with only 10–15% of all cases identified globally [20]. In a recent study in the Greek rural population aged 40–65, it was verified that the respiratory symptoms of smokers were underestimated and thus not diagnosed as COPD cases, suggesting that the actual COPD prevalence in Greece may be even higher [11].

According to the 2022 GOLD Report, the COPD prevalence globally is 11.7% based on the GOLD criteria [21]. The most recent systematic review and modelling study using data from 1990 until the end of 2019 showed that the global prevalence of COPD is 10.3% with a high variability depending on the geographic area and the corresponding socioeconomic status of the area [22]. Based on this study, the global COPD prevalence for the median age groups matching the male and female participants of our study population (56 and 54, respectively) would be 17.9% and 6%, respectively. However, in our study, the COPD prevalence based on COPD-GOLD criteria in males was 8.91% and in females was 9.09%. The overall COPD prevalence was 8.99% in our population, which is within the range reported by Greek studies within the previous two decades, which reported values between 5.6% and 18.4% [23,24,25]. We found an overall COPD prevalence that was nearly half of that reported in the last study performed in Thessaly (18.4%), which did not, however, involve insular primary health centers [25]. There has been no other study using spirometry to evaluate COPD prevalence in insular Greece to compare our results. The results of COPD-PS in our study population showed a self-reported COPD prevalence of 18.8%, which was double the actual GOLD-based prevalence. This could be a useful tool for screening in the insular community since, based on our results, the NPV was 0.83 and the specificity 92.21%, thus qualifying this as an apt COPD exclusion tool. The sensitivity of 14.29% and specificity of 92.21% are in agreement with a previous Greek study that validated COPD-PS in a primary-care setting in mainland Northern Greece [26]. COPD development has been shown to be inversely correlated to Mediterranean diet adherence [27]. In our group, there was no difference in the MedDietScore questionnaire results between COPD and non-COPD participants. MedDietScore indicating moderate to high adherence to Mediterranean Diets corresponds to scores over 31/55 [9,28]. Based on the above, in our sample, both COPD and non-COPD participants had a marginally moderate adherence to Mediterranean Diet, thus its beneficial effects on COPD development were not present in our group.

The self-reported prevalence of OSA was found to be 27.09%, similar to what was found in two previous reports by our research group that were performed as telephone surveys and assessed self-reported OSA community prevalence [1,3]. The first study was performed in the insular population of 41 Greek islands (other than the Sporades insular complex) of the Aegean and Ionian Pelagos with a self-reported prevalence of 27.29%. The latter studied a mainly urban population comprising four cities (Larissa, Volos, Trikala, and Karditsa) and three islands of Sporades of the Prefecture of Thessaly in Greece and found a self-reported OSA prevalence of 25.49%. The OSA prevalence we found in our study was similar to what has been found internationally: 26% in the USA, 25% in Switzerland, and 24.3% in Norway [29,30,31]. Regarding the daytime sleepiness as indicated by the ESS, we report here a self-reported prevalence of 27.66%, nearly twice the prevalence that was previously found in Greece in the telephone surveys concerning the population of the Greek islands (16.14%) and Thessaly prefecture (6.37%). Our results are higher compared to similar studies in Switzerland (12%), Norway (8.9%), and Sweden and Iceland (13.1%) [30,31,32]. However, our results are not higher than what has been reported in a similar study in Canada (33%) [33]. Self-reported OSAS prevalence (that is, a participant found at high risk in both BQ and ESS) was found in 9.48% (10.53% in men and 8.40% in women). In a recent systematic review that assessed the prevalence of OSAS in the general population, this value was estimated within a range of 6%–17%, therefore our results agree with the published literature [34].

A strength of our study was that we provide the first detailed COPD prevalence and OSAS self-reported prevalence in a significant fraction (approximately 8%) of the population of a non-profit line Greek island. Our data can serve as a tool for designing health-promotion strategies tailored to the needs of such populations, which are numerous in Greece, and improving their overall health. However, an important limitation of our study was that the study population consisted of volunteering participants, which likely resulted in selection bias. The recruitment of slightly less than 10% of the population aimed specifically at increasing the reliability and applicability of our data in the context of the selection bias limitation of the study.

## 5. Conclusions

In this study, we report a high prevalence of COPD, OSA, and daytime sleepiness. Considering the underdiagnosis of COPD and OSA and the fact that COPD is among the leading causes of mortality, the implementation of Health Promotion Programs that will raise the awareness of the local general population is important. Reinforcing the education of health professionals to achieve early diagnosis and treatment of the above-mentioned diseases is also important. Finally, we confirmed in our study population that COPD-PS can be effectively used as a COPD exclusion tool in the setting of small Primary Healthcare Centers in Greece.

## Figures and Tables

**Table 1:** Demographic Characteristics of the Study Participants.

	Males	Females	*p*
Gender	115 (48.73%)	121 (51.27%)	
Age	56 (42, 73)	54 (38.50, 67.50)	0.205
BMI	27.70 (24.68, 30.52)	25.61 (22.59, 29.34)	0.005
Smoking Status	Smokers: 48 (41.74%) Ex-Smokers: 53 (46.09%) No Smokers: 14 (12.18%)	Smokers: 51 (42.50) Ex Smokers: 19 (15.83%) No Smokers: 50 (41.67%)	<0.001
Packyears	35 (11, 50)	13.50 (7, 25)	<0.001
MedDietScore	33 (29.75, 36)	32 (29, 35)	0.181

Note: Data are presented as median and IQR or n (%). One female participant did not provide information about her smoking status. Bold *p* values denote significance.

**Table 2:** Participants at high risk for COPD according to COPD-PS.

	COPD-PS High	COPD-PS Low	*p*	COPD	No COPD	*p*
Age	69 (60, 76.50)	50 (37, 64.25)	<0.001	64 (49, 72.50)	52 (38, 64.75)	0.021
BMI	27.75 (24.57, 32.11)	26.27 (23.40, 29.49)	0.056	26.42 (22.07, 28.66)	26.86 (23.92, 30.35)	0.237
Smoking Status	Smokers: 19 (44.18%) Ex Smokers: 21 (48.84%) No Smokers: 3 (6.98%)	Smokers: 79 (41.79%) Ex Smokers: 50 (25.46%) No Smokers: 60 (31.75%)	0.001	Smokers: 9 (56.25%) Ex Smokers: 5 (31.25%) No Smokers: 2 (12.50%)	Smokers: 74 (43.02%) Ex Smokers: 55 (31.98%) No Smokers: 43 (25.00%)	0.464
Packyears	45 (20, 66)	16 (7, 35)	<0.001	34 (19.50, 81.50)	22 (9.88, 45)	0.036
MedDietScore	33.50 (30.25, 36)	32 (29, 35)	0.259	33 (29.50, 36)	32 (29, 35)	0.661

Note: Data are presented as median and IQR or n (%).

**Table 3:** High-risk participants for BQ, EDS, and BQ+ESS.

	BQ High	BQ Low	*p*	ESS High	ESS Low	*p*	BQ+ESS High	BQ+ESS Low	*p*
Age	66 (55, 74)	50 (37, 64)	<0.001	63 (49, 70)	51 (38, 66)	0.009	71.5 (60.25, 79)	53 (38, 66.25)	<0.001
BMI	30.86 (27.43, 33.63)	25.50 (23.13, 28.08)	<0.001	27.46 (23.48, 31.71)	26.49 (23.46, 29.41)	0.368	31.63 (26.48, 33.29)	26.30 (23.43, 29.36)	<0.001
Smoking Status	Smokers: 14 (22.22%) Ex-Smokers: 29 (46.03%) No Smokers: 20 (31.75%)	Smokers: 84 (49.70%) Ex-Smokers: 42 (24.85%) No Smokers: 43 (25.45%)	<0.001	Smokers: 23 (35.38%) Ex-Smokers: 25 (38.46%) No Smokers: 17 (26.16%)	Smokers: 75 (43.62%) Ex-Smokers: 46 (26.74%) No Smokers: 46 (26.74%)	0.239	Smokers: 5 (22.73%) Ex-Smokers: 12 (54.54%) No Smokers: 5 (22.73%)	Smokers: 92 (43.80%) Ex-Smokers: 59 (28.10%) No Smokers: 59 (28.10%)	0.032
Packyears	35 (12, 52)	20 (7.5, 38)	0.025	25.50 (11, 50)	20 (7, 39)	0.032	50 (21.5, 96)	20 (7.5, 39)	<0.001
MedDietScore	33 (29, 36)	32 (29, 35)	0.202	32 (29, 35)	32 (29, 35)	0.850	34.50 (31, 37.25)	32 (29, 35)	0.035

Note: Data are presented as median and IQR or n (%).

## Data Availability

Data are available from the corresponding author upon reasonable request.
